# Deterioration of Visual Acuity after Brachytherapy and Proton Therapy of Uveal Melanoma, and Methods of Counteracting This Complication Based on Recent Publications

**DOI:** 10.3390/medicina59061131

**Published:** 2023-06-12

**Authors:** Jakub Jarczak, Izabella Karska-Basta, Bożena Romanowska-Dixon

**Affiliations:** 1 Department of Ophthalmology, Division of Ophthalmology and Ocular Oncology, Faculty of Medicine, Jagiellonian University Medical College, 31-501 Krakow, Polandromanowskadixonbozena1@gmail.com (B.R.-D.); 2 Jagiellonian University Medical College, Doctoral School of Medical and Health Sciences, 31-530 Krakow, Poland

**Keywords:** uveal melanoma, radiation therapy, brachytherapy, proton therapy, visual acuity, vision deterioration, radiation complications

## Abstract

Uveal melanoma (UM) is the most common primary intraocular malignancy in adults. The eyeball is the most common extracutaneous location of melanoma. UM is a huge threat to a patient’s life. It metastasizes distantly via blood vessels, but it can also spread locally and infiltrate extraocular structures. The treatment uses surgical methods, which include, among others, enucleation and conservative methods, such as brachytherapy (BT), proton therapy (PT), stereotactic radiotherapy (SRT), stereotactic radiosurgery (SRS), transpupillary thermotherapy (TTT) and photodynamic therapy. The key advantage of radiotherapy, which is currently used in most patients, is the preservation of the eyeball with the risk of metastasis and mortality comparable to that of enucleation. Unfortunately, radiotherapy very often leads to a significant deterioration in visual acuity (VA) as a result of radiation complications. This article is a review of the latest research on ruthenium-106 (Ru-106) brachytherapy, iodine-125 (I-125) brachytherapy and proton therapy of uveal melanoma that took into account the deterioration of eye function after therapy, and also the latest studies presenting the new concepts of modifications to the applied treatments in order to reduce radiation complications and maintain better visual acuity in treated patients.

## 1. Introduction

Uveal melanoma (UM) is the most common primary intraocular malignancy in adults, with the highest incidence of 5–10 per million Whites per year. The eyeball is the most common extracutaneous location of melanoma [1,2,3,4,5,6,7,8]. Usually, it is located in the choroid (90%), and less often in the ciliary body (6%) and iris (4%) [9]. The peak of incidence falls in the sixth to the seventh and, to a lesser extent, the third decade of life [2]. Uveal melanoma metastasizes distantly via blood vessels, most often to the liver (93%), lungs (24%) and bones (16%). It can also spread locally and infiltrate extraocular structures. Extraocular infiltration is associated with a higher risk of distant metastases and worse prognosis. Melanoma metastases are found in most patients at the time of their death [10,11]. They can even be detected several years after successful treatment of the primary cancer, which may be associated with their presence, but also the inability to detect them at the time of therapy [1]. The prognosis in patients with confirmed distant metastases is very poor, and the 1- and 3-year survival rates are approximately 21% and 4%, respectively [12]. Unfortunately, there are currently no effective treatments for distant metastases, which are less susceptible to chemotherapy than skin melanoma metastases [13]. Recently, there have been studies presenting therapies with the use of Tebentafusp and giving hope for improving this situation [14]. This bispecific glycoprotein 100 peptide-HLA-directed CD3 T-cell engager was approved by the Food and Drug Administration (FDA) in 2022 for patients with unresectable or metastatic uveal melanoma. In the same year, it received a positive response from the European Union Committee for Medicinal Products for Human Use for the treatment of uveal melanoma [15]. UM shows specific chromosomal abnormalities. The most significant is the complete or partial loss of chromosome 3. Other common genetic changes are deletions in 1p, 6q, 8p and 9p, and additions in 1q, 6p and 8q [16]. There have been publications describing familial cases of UM belonging to the familial BAP1-associated cancer predisposition syndrome inherited in an autosomal dominant manner [16,17].

**Figure 1:**
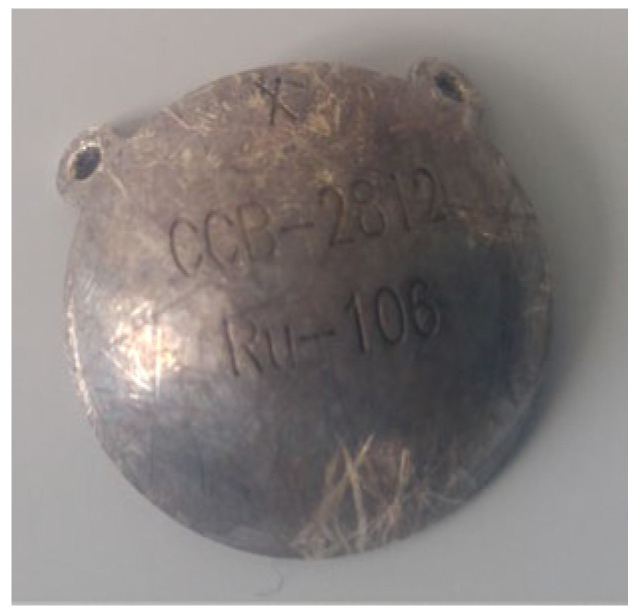
Ruthenium-106 applicator after several dozen uses.

**Figure 2:**
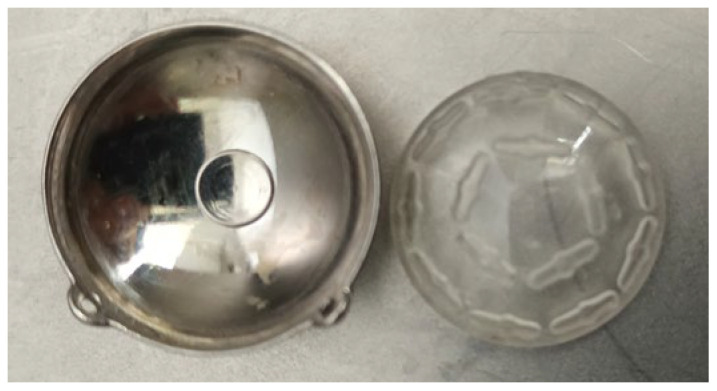
Iodine-125 applicator (metal shell and polymer insert with spaces for iodine seeds).

**Figure 3:**
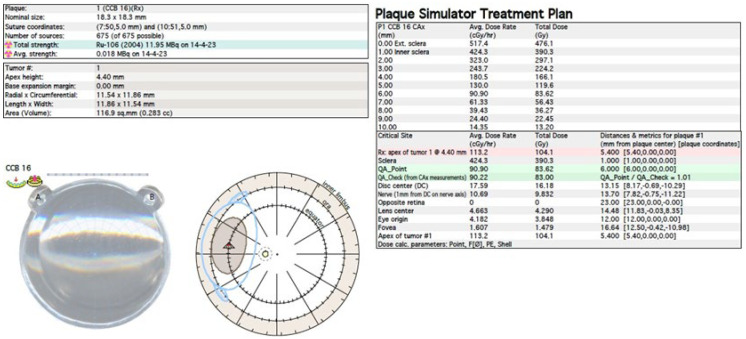
Ruthenium-106 brachytherapy planning system in Plaque Simulator 6.8.5—planning cards.

**Figure 4:**
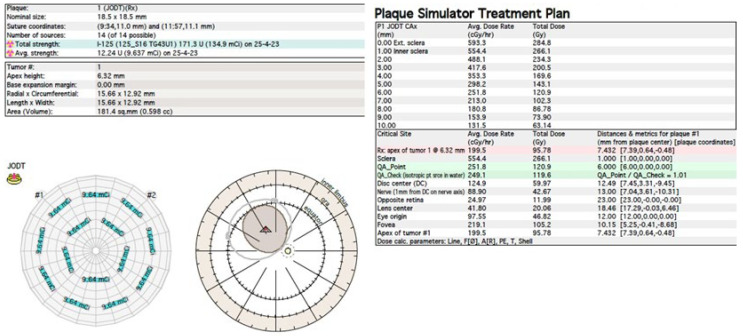
Iodine-125 brachytherapy planning system in Plaque Simulator 6.8.5—planning cards.

The treatment uses surgical methods, which include, among others, enucleation, and conservative methods, such as brachytherapy (BT), proton therapy (PT), stereotactic radiotherapy (SRT), stereotactic radiosurgery (SRS), transpupillary thermotherapy (TTT) and photodynamic therapy. Many factors are taken into account when planning a treatment strategy, including the size of the tumor, its location and the general condition of the patient. Initially, the treatment of choice in uveal melanoma was enucleation. Over time, brachytherapy began to play a dominant role [1,4,18]. The increase in the popularity of brachytherapy was largely influenced by the publication of the results of the Collaborative Ocular Melanoma Study (COMS), which showed the lack of an advantage of enucleation over brachytherapy in terms of distant metastases and survival in the case of small- and medium-sized tumors [3,19]. In a COMS study, tumor size was defined as follows: small: 1.5–2.4 mm in height and 5–16 mm in diameter; medium: 2.5–10 mm in apical height and ≤16 mm in diameter; and large: >10 mm in apical height and >16 mm in diameter; these terms will be used to describe tumors of these dimensions in the remainder of this article, unless otherwise noted [3,11]. Brachytherapy has gradually become the most commonly used treatment for choroidal melanoma. It involves suturing a plate containing a radioactive isotope to the sclera in the place where the base of the tumor is located. After a few days to inactivate the tumor, the applicator is removed from the sclera [1,20]. In Europe and Asia, the most commonly used radioactive isotope is ruthenium-106 (Ru-106), which emits beta radiation (Figure 1). In North America, iodine I-125 (I-125) is dominant, which emits gamma radiation (Figure 2). Less frequently used isotopes are Pd-103, Sr-90, Ir-192, Cs-131, Au-198 and others. There are different sizes and shapes of plates, e.g., with a notch for the optic nerve, dedicated to the treatment of tumors located at the optic disc. The time of exposure to radiation is several days and depends on the thickness of the tumor and the activity of the applicator. Treatment planning is carried out using specialized computer systems (Figure 3 and Figure 4) [9,18,20]. Local tumor control, depending on the source of radiation, ranges from 59–98% for ruthenium-106 [21] to 76–100% for iodine-125 [22]. Brachytherapy can also be combined with other methods, e.g., TTT, and this combination is called “sandwich therapy” [1,18,23].

**Figure 5:**
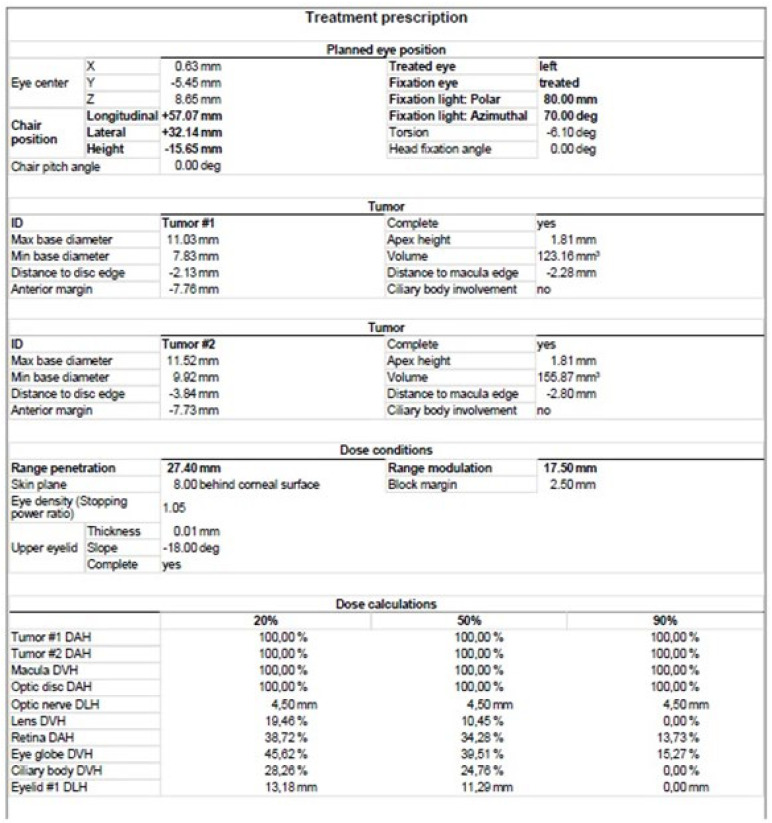
Proton therapy planning system in Eclipse Ocular Proton Planning version 13.5.01−planning card.

Another method of irradiation is radiation therapy from an external source. Among this group, proton therapy is the most common. It is also a newer and much less frequently used method than brachytherapy. It uses a beam of protons, which, owing to the Bragg peak phenomenon, allows the irradiation of the tumor mass to an equal extent while sparing the surrounding healthy tissues. Its development gave hope for a significant reduction in post-radiation complications and thus better eye function after treatment. Before using this method, the patient first undergoes a surgical procedure of the placement of a few tantalum markers by sutures to the outer sclera to localize the base of the tumor, which is visualized by transillumination. Measurements of the distance between the structures of the eyeball are also performed using ophthalmic imaging techniques, such as optical coherence tomography (OCT), ultrasonography (USG) and fundus photography. As in the case of brachytherapy, treatment planning is carried out using specialized computer systems (Figure 5) [1,18,20]. Proton therapy has excellent local tumor control of 95% [24].

Stereotactic radiotherapy (SRT) and stereotactic radiosurgery (SRS), which also represent external-source radiotherapy, have gained popularity in the treatment of uveal melanoma in recent years. Irradiation of the tumor can be performed in a single session with a high dose of photons (SRS) or in a fractionated form (SRT) [11,20,25,26,27,28]. The main advantages of these methods include their minimal invasiveness, excellent local tumor control, relatively wide availability and low costs [20,25,26,28,29]. Some researchers have indicated their disadvantages in the form of lower precision and the associated higher number of post-radiation complications, mainly secondary glaucoma and enucleation compared with brachytherapy and proton therapy [11,20,30]. Other studies report no significant differences in the rate of complications between these therapies [18].

Most of the studies on choroidal melanoma therapy published so far have focused on comparing patient survival, risk of distant metastases and local tumor control after various treatments. Less attention has been paid to the problem of visual acuity (VA) deterioration after therapy. This is understandable, as the priority is to save the patient’s life. Nevertheless, the deterioration of eye function caused by radiation complications in the form of damage to structures such as the macula, optic disc and lens is a serious problem [9,11]. After brachytherapy, 49% of eyes experienced vision loss within three years and 68% within ten years. In addition, 45% of patients were qualified as blind (20/200 or worse) within three years post-treatment [31].

In the following part of the article, we present the latest chosen research on Ru-106 brachytherapy, I-125 brachytherapy and proton therapy of uveal melanoma that took into account the deterioration of eye function after therapy, and also the latest studies presenting the new concepts of the modification of the applied treatment in order to reduce radiation complications and maintain better visual acuity in treated patients. We took into account articles published in English and available in the PubMed database from 2019 to the present. It should be emphasized that this article is a review, not a systematic review, and was not prepared in accordance with the Preferred Reporting Items for Systematic Reviews and Meta-Analyses (PRISMA) guidelines. We believe that paying more attention to the problem of vision deterioration, combined with the search for new solutions that could counteract this problem, will allow the introduction of modifications to clinical practice that will minimize the deterioration of visual acuity while maintaining the effectiveness of treatment.

## 2. Ru-106 Brachytherapy

**Figure 6:**
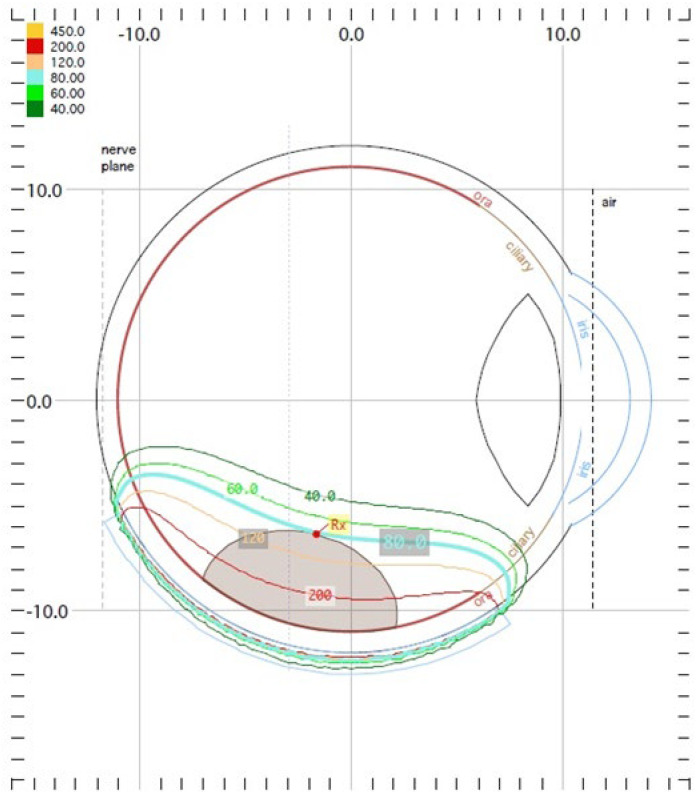
Ruthenium−106 brachytherapy planning system in Plaque Simulator 6.8.5−dose distribution presentation.

Ru-106 brachytherapy emits beta radiation and is used for tumors up to 5 mm in height due to the limited range of radiation penetration, but in many centers, it is successfully used to treat thicker tumors (Figure 6). Therefore, ruthenium is intended for the treatment of small and some medium-sized tumors [20,32].

The use of Ru-106 for thicker tumors was explored by Cennamo et al., who enrolled 350 eyes with small- and medium-sized tumors (measuring up to 6.5 mm in apical height) after Ru-106 plaque brachytherapy for uveal melanoma with a total dose of 100 grays (Gy) to the tumor apex in a retrospective study. Radiation complications were found in 63% of patients: 38% showed radiation maculopathy, 11% had optic neuropathy and 14% developed cataracts. At baseline, the mean best*corrected visual acuity (BCVA) according to the Early Treatment of Diabetic Retinopathy Study (ETDRS) in affected eyes was 0.32 ± 0.30 logarithm of the minimum angle of resolution (logMAR). The mean follow-up was 4 years (3 months to 9 years). The posttreatment BCVA was 0.40 ± 0.25 logMAR. The patients who presented radiation-related complications showed reduced visual acuity (0.7 ± 0.85 logMAR) [33].

Mirshahi et al. conducted a study in which Ru-106 was also used to treat tumors thicker than 6 mm. They analyzed the medical records of 234 patients undergoing Ru-106 plaque brachytherapy for UM. The target dose to the apex was 100 Gy. The median follow up was 54.2 months (range: 6–194.5 months). Visual acuity (logMAR) was 0.673 ± 0.70 at baseline and 0.963 ± 0.80 at the final examination (excluding enucleated eyes). VA was >20/200 at the baseline in 60.3% of patients and at the last examination in 44.2% of patients. VA was >20/40 at the baseline in 28.6% of patients and at the end of follow up in 18.1% of patients. The following radiation complications were found in patients: 108 eyes (46.2%) developed cataracts, and radiation retinopathy was found in 58.1%, radiation maculopathy in 29.9% and radiation papillopathy in 20.1% of patients. Radiation papillopathy was associated with the total scleral dose (B = 0.001, 95% CI: 0.001 to 0.001, *p*-value = 0.036) and distance to the optic nerve head (B = −0.292, 95% CI: −0.226 to −0.358, *p*-value <0.001). After adjusting for baseline VA, the final logMAR BCVA was associated with radiation maculopathy and papillopathy, and the rate of legal blindness (BCVA < 20/200) was 44.8%. Interestingly, there was no significant association between the final VA and different tumor dimension and location groups [34].

O’Day et al. decided to assess the long-term visual outcomes after ruthenium plaque brachytherapy for posterior choroidal melanoma. Anterior tumors involving the iris and ciliary body were excluded from this study. A total of 219 patients with posterior choroidal melanoma were treated with ruthenium plaque brachytherapy between January 2013 and December 2015. The baseline visual acuity was ≥6/12 in 168 (76.7%), 6/18–6/60 in 43 (19.6%) and <6/60 in 8 (3.7%) eyes. A dose of 80 (9.1%), 100 (89.5%) or 120 (1.4%) Gy to the apex was prescribed. The median follow up was 56.5 months (12–81 months). The final visual acuity was ≥6/12 in 97 (44.3%) patients, 6/12 to 6/60 in 57 eyes (26.0%) and <6/60 in 55 (25.1%) eyes. Ten (4.6%) eyes were enucleated. The most common radiation complication was radiation maculopathy, which affected 53 (24.2%) patients. Statistical analysis revealed that close proximity to the optic disc and fovea and a large or notched plaque type were associated with poorer final visual acuity (<6/12) upon long-term follow up. A distance of 5.0 mm or more from the optic nerve and fovea was the most significant positive prognostic factor in terms of preservation of vision. The results also showed that using smaller plates allowed maintaining better vision than using larger ones [35]. However, it should be noted that the reduction in plaque size was limited by the size of the tumor.

## 3. I-125 Brachytherapy

**Figure 7:**
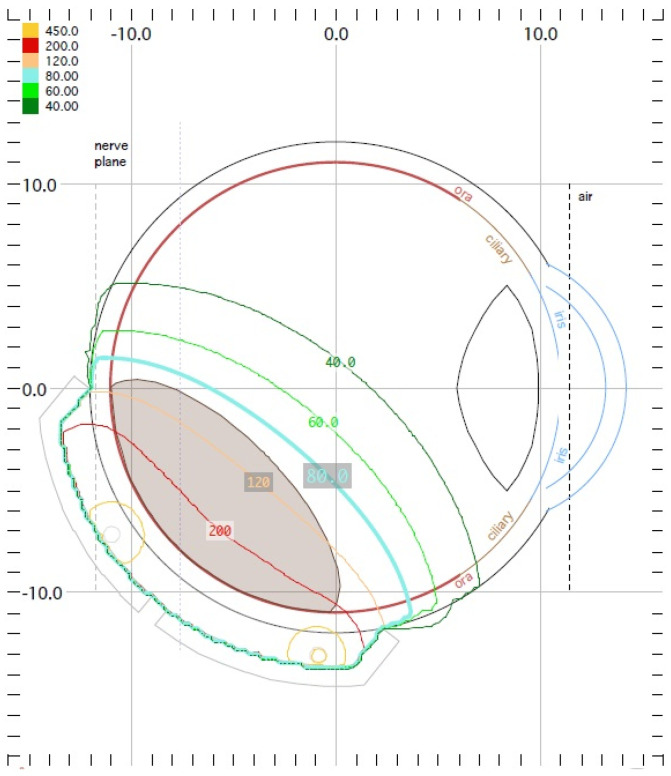
Iodine−125 brachytherapy planning system in Plaque Simulator 6.8.5−dose distribution presentation.

I-125 brachytherapy can be used for larger tumors than Ru-106, but is recommended for tumors up to 10 mm in height [36]. The treatment of larger tumors is possible due to the gamma radiation emitted by I-125, which has a longer range than the beta radiation from Ru-106 (Figure 7). Unfortunately, it is also associated with a higher dose of radiation delivered to other structures of the eye and damage to them [20,32].

The study by Oare et al. was mainly devoted to complications resulting from the radiation of healthy eye structures. They analyzed the relationship between the dose received by the eye structures (macula, optic disc and lens) and ocular complications, including visual impairment, after I-125 brachytherapy. The retrospective study included 52 patients treated from 2005 to 2019 with an average follow-up time of 4.0 years (median: 3.6; range: 0.4–13.5 years). Among the many interesting results, the most important seemed to be that, after follow up, 65% of patients presented radiation retinopathy, 65% developed cataracts, 85% had at least a mild change in vision, 40% had moderate vision decline (>5 Snellen lines lost) and 31% eyes became blind (VA = 20/200 or worse) [31].

In another study, Tamplin et al. decided to define the temporal relationship between vascular and neuronal abnormalities in radiation retinopathy in 25 patients after I-125 brachytherapy due to choroidal melanoma. They used ophthalmic imaging methods, such as OCT, OCT angiography and digital fundus photography. The average follow-up time was 33 months. The level of structural abnormality was determined by interocular asymmetry compared with normal subject asymmetry. However, in their study, they also took into account the effect of treatment on visual acuity, which is of interest to us. The difference in the field of vision of the patients undergoing the treatment was also examined. The degree of structural abnormality was determined by the intereye asymmetry compared with normal subject asymmetry. Abnormal visual acuity (logMAR), defined as a loss of two or more Snellen lines since the time of diagnosis, was observed in 17% of patients tested at or after 2 years after brachytherapy. An abnormal visual field, defined by a mean deviation and/or pattern SD of *p* ≤ 5%, was observed in 67% of patients tested at or after 2 years after brachytherapy. The study provided results that indicated that radiation-induced ischemia was a primary early symptom of radiation retinopathy preceding vision deterioration [37].

Dalvin et al. undertook a very ambitious goal consisting of developing a nomogram for the prediction of visual acuity outcomes following plaque radiotherapy for uveal melanoma. A total of 1131 patients were treated with I-125 plaque radiotherapy and prophylactic intravitreal bevacizumab injections (1.25 mg/0.05 mL) at the time of plaque removal and again at 4-month intervals for a duration of 2 years in order to prevent the development of macular edema after plaque radiotherapy. Patients treated from 2000 to 2018 were included in this retrospective study. The average baseline BCVA (logMAR) was 0.35 (Snellen equivalent 20/50). The mean total radiation dose to the tumor apex was 70 Gy. The average follow-up time after treatment was 40 months. The mean BCVA at the last follow-up was 1.02 (Snellen equivalent 20/200) and poor visual acuity worse than 20/200 was presented by 33% of patients. The authors assigned an appropriate sum of points to the various clinical and treatment risk factors. In their opinion, the most important risk factors for a poor visual acuity outcome were: subretinal fluid involving four quadrants, tumor thickness of >4 mm, baseline visual acuity of ≤20/30, race different than Caucasian, specific tumor shapes, insulin-dependent diabetes, largest tumor basal diameter of >11 mm, radiation dose rate to tumor base of ≥164 cGy/hour and abnormal foveolar status in OCT examination at baseline. The developed nomogram could predict the visual acuity outcome after plaque radiotherapy and prophylactic intravitreal injections of bevacizumab [38]. The authors also emphasized the great role of intravitreal injections of bevacizumab, recalling their previous study, in which the median BCVA 48 months after plaque radiotherapy for patients managed with bevacizumab versus controls was 20/70 versus counting fingers (*p* < 0.001) [39]. We will cover bevacizumab injections in more detail later in this article.

## 4. Ru-106 Brachytherapy vs I-125 Brachytherapy

In recent years, studies comparing brachytherapy with iodine-125 and ruthenium-106 have also been published. We present some of these studies in which the quality of vision after therapy was assessed.

Ghassemi et al. carried out a retrospective, non-randomized comparative case series study. Out of 35 patients with choroidal melanoma, 15 were treated with I-125 and 20 with Ru-106 plaques from 2013 to 2017. The visual acuity (logMAR) before treatment was 0.8 ± 0.2 for patients treated with Ru-106 and 1.3 ± 0.3 for those treated with I-125. The last registered visual acuities (logMAR) after a follow up of a mean of 29.7 months (range: 15–54 months; SD: 9.0 months) were 0.90 ± 0.3 and 1.4 ± 0.2 for patients after Ru-106 and I-125 brachytherapy, respectively. There was no significant difference in vision deterioration and ocular complications between the two analyzed groups [40].

A huge systematic review containing an analysis of 103 studies on patients treated with plaque brachytherapy from 2005 to 2020 was prepared by Buonanno et al. The purpose of the study was to provide an overview of local tumor control and treatment-related toxicity after plaque brachytherapy for uveal melanoma. Among many other important conclusions, such an extensive analysis of publications from 16 years presented a significant reduction in the metastasis rate and enucleation events and an increased disease-specific survival rate in patients receiving therapy in which treatment planning systems (TPS) were used. Moving on to the complications, the analyzed studies showed that using Ru-106 had a lower risk for ocular morbidity than other plaque sources owing to its dosimetric characteristics. Ru-106 treatment, compared with I-125 brachytherapy, showed reduced radiation-related morbidity occurrence for the pooled rate of cataracts, optic neuropathy and neovascular glaucoma; however, the scleral necrosis risk was higher for Ru-106 compared with I-125 and Pd-103 plaques [9].

Zemba et al. also focused on ocular complications of radiotherapy in uveal melanoma. Their systematic review contained 78 articles dating from 1996 to 2021. In addition to brachytherapy and proton beam therapy, their review also included a less frequently used stereotactic radiosurgery. Their results showed that a final VA of less than 20/200 was reported in 23–87% of cases after plaque brachytherapy, in 33–86% of cases after proton beam therapy and in 60–65% of cases after stereotactic radiosurgery. They also presented risk factors for the development of certain post-radiation complications, depending on the radiotherapy used, with differentiation between iodine and ruthenium brachytherapy too. Their analysis confirmed earlier reports about the critical role of the dose absorbed by the eye structures critical for vision, such as the macula and optic nerve, in visual acuity after therapy. The role of other risk factors in the deterioration of vision after treatment, such as increased tumor thickness, tumor location close to the macula or optic nerve and many others was also emphasized [11].

## 5. Proton Therapy

**Figure 8:**
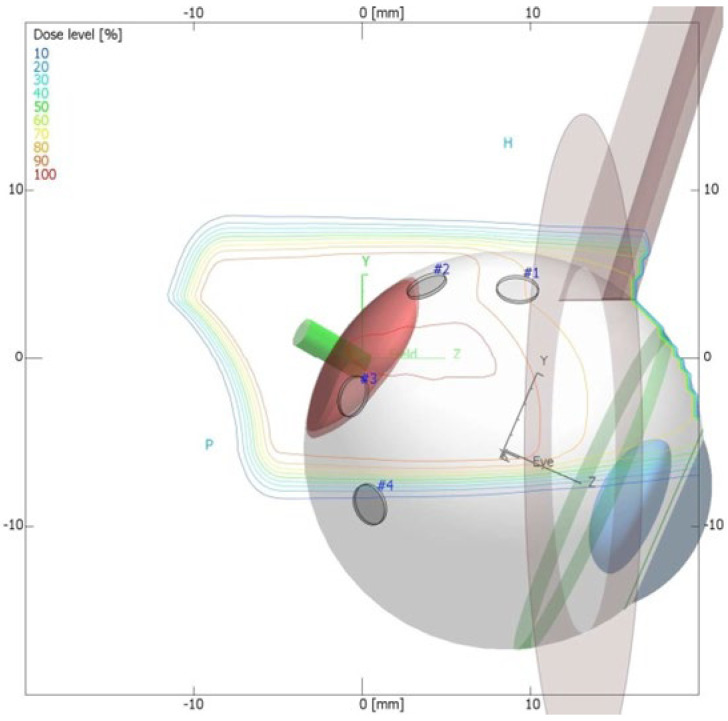
Proton therapy planning system in Eclipse Ocular Proton Planning version 13.5.01−dose distribution presentation.

The possibility of using proton therapy in the treatment of intraocular tumors gave great hope for avoiding radiation complications. Owing to the Bragg peak phenomenon, it allows the irradiation of the tumor mass to an equal extent while sparing the surrounding healthy tissues (Figure 8). Over time, it was found that this was not an ideal therapy and also had its drawbacks [18,20]. Nevertheless, it is constantly evolving, and it is definitely worth taking a closer look at the latest research on it.

Researchers from the Netherlands conducted an interesting retrospective analysis of the effects of proton beam radiotherapy on their compatriots treated in Switzerland from 1987 to 2019. The tumors of these 103 patients enrolled in the study were too large or localized too close to the optic nerve to be treated with brachytherapy. Proton beam therapy was offered to patients if the height of the tumor was >7 mm and diameter was >16 mm, or the involvement of the circumference of the optic disc was >1/3. Modified EYEPLAN planning software was used to plan proton beam therapy. The median follow-up was 7 years. All patients received 60 cobalt gray equivalent (CGE) in four fractions during the four subsequent days. The result of Marinkovic et al.’s study revealed that, 5 years after treatment, 79% of patients lost their vision or had severe (<0.1; Snellen <6/60; logMAR >1.0) visual impairment. Moderate (0.1 to <0.3; Snellen 6/60 to <6/20; logMAR 1 to >0.5) and mild (<0.5; Snellen 6/20 to <6/12; logMAR 0.5 to >0.3) visual impairment was observed in 4% and 6% of the patients, respectively. The most important factors responsible for the decrease in visual acuity were the size of the tumor, its localization and radiation dose. The portion of radiation of 30 CGE (cobalt gray equivalent) absorbed by the macula, optic disc and optic nerve was clearly related to severe visual impairment [41]

Espensen et al. conducted dose–response modeling to examine the relationships between the dose delivered to healthy tissue, the occurrence of VA deterioration and various radiation-induced toxicities after treatments with proton therapy. It was a retrospective study from a single referral center in France that involved 1243 patients treated with hypofractionated proton therapy for choroidal melanomas between 1991 and 2015. From this group, vision deterioration information was available for 1020 patients and information about late complications was available for 991 patients. The tumor dose was 52 Gy in four equal fractions. EyePlan version 3.06 was used as the treatment planning system. The researchers used dose volume histograms (DVHs) or dose surface histograms (DSHs) for each of the analyzed anatomic structures extracted from the EyePlan. Upon post-treatment follow-up for a minimum of 5 years (median potential follow-up time was 6.1 years), the patients were assessed, including, inter alia, the best corrected visual acuity using the Snellen scale, which was converted into the logMAR for analysis purposes. Visual acuity deterioration was defined as a loss of 0.3 logMAR or more compared with the baseline measure. The average baseline VA (logMAR) was 0.4 with a median (IQR, interquartile range) of 0.2–1.0. The average last VA (logMAR) was 1.6, with a median (IQR) of 0.4–2.0. Before treatment, 53% of the qualified patients had VA at the level of logMAR ≤0.5. After at least 5 years, only 29.4% of patients maintained this quality of vision. The researchers proved that the near-maximum dose to the macula showed the strongest correlation with VA deterioration, but tumor height was associated with the risk of VA deterioration even stronger than the maximum dose to the macula. They also described the risk of visual acuity deterioration and number of radiation complications, depending on the dose received by other anatomical structures of the eye. It has been shown that VA deterioration depends on the dose to a range of structures, while specific radiation complications were related to the dose metrics received by individual structures [42].

Another study was unique because the patients were Asians, among whom uveal melanoma is much less common than in Caucasians. Its prevalence in Asia is estimated to be 0.2–0.6 cases per one million individuals [43,44]. Korean researchers led by Su-Kyung Jung conducted a study involving 40 patients who received PT for choroidal melanoma in 2009–2016 in a single institution in the Republic of Korea. The median follow-up duration was 32 (12–82) months. EYEPLAN was used as the treatment-planning system. The dose prescription was 60–70 CGEs in five fractions. BCVA examinations were performed as part of the ophthalmic examination during post-treatment follow-up. A pretreatment BCVA of ≥20/40 was selected to find the prognostic factors affecting VA. The vision loss was defined as a final BCVA under 20/80. The pretreatment BCVA was ≥20/40 in 70% of patients, while in the other 30%, it was <20/40. The analysis of the prediction factors of the final BCVA was performed in the patient who had a baseline BCVA of ≥20/40, because patients who had a baseline BCVA of <20/40 abruptly had a poor VA after PT. The BCVA in the treated eyes was classified as ≥20/50 and <20/50 or worse during the total follow-up period after PT. The created groups were compared in terms of factors affecting the deterioration of vision. The final BCVA of around one-third of the PT-treated choroidal melanoma patients with good pretreatment BCVA was maintained (>20/40). The study showed that tumor size, the tumor’s distance from the optic disc or the fovea, but also the volume of the macula (30 CGEs), optic disc (30 CGEs) and retina (30 CGEs) receiving radiation, could be expected to affect the final VA. The main cause of vision loss was intraocular bleeding [44].

A summary of the most important features of the analyzed therapies is presented in Table 1.

## 6. Concepts for Improving Brachytherapy and Proton Therapy in Terms of Preventing Post-Radiation Complications

Despite the great progress in the field of uveal melanoma radiotherapy in the last few decades, radiation complications often result in a diametric worsening of visual acuity, complete blindness of the treated eye or removal of the eyeball, remaining a serious problem [20,21]. Therefore, the search for a solution enabling better protection of healthy eye structures to avoid radiation complications while maintaining, or even improving, the efficiency of tumor inactivation is underway. Recent publications on some such solutions are presented below.

According to many previous studies, the frequency of complications after brachytherapy increases with the increase in the dose received by the healthy structures of the eye [11,31]. Fili et al. published a study aiming to investigate if a threshold for increased disease-related mortality could be identified within the clinical range of prescribed doses and dose rates among patients treated with ruthenium-106 or iodine-125 brachytherapy for choroidal melanoma. However, based on the results of that study, we can draw conclusions regarding the visual acuity deterioration after brachytherapy. A total of 1238 patients treated at St. Erik Eye Hospital in Sweden from 1996 to 2016 were included. Patients were distributed into decile groups based on the apex dose and dose rate, respectively. These groups were then compared in terms of many parameters, primarily melanoma-related mortality. The average radiation dose at the tumor apex ranged from 73.0 Gy in the first decile to 108.6 Gy in the tenth. The mean radiation dose rate at the tumor apex ranged from 0.5 Gy/hour in the first decile to 2.8 Gy/hour in the tenth. The analysis of the results showed that there were no increased risks for choroidal melanoma-related mortality after brachytherapy with decreasing doses between 108.6 and 73.0 Gy, or with decreasing dose rates between 2.8 and 0.5 Gy/hour [45]. Considering also the results of the systematic review from 2017, which showed no statistical significance of a higher dose with regard to better local tumor control, it seems reasonable to conduct further research on reducing the used dose to reduce the deterioration of visual acuity after treatment [22].

The replacement of the vitreous body with silicone oil prior to brachytherapy is one of the strategies to reduce post-radiation complications which may lead to the need for enucleation [46]. The use of silicone oil shielding with iodine-125 plaques has previously been reported to protect sensitive, critical structures in the past. Other substances were also tested, but silicone oil was the best solution [47,48]. Yang et al. decided to determine if the use of palladium-103 (Pd-103) would improve the shielding effectiveness of silicone oil due to the strong energy dependence of the photoelectric effect. The Geant4 v10.0322 Monte Carlo radiation transport toolkit was used in that study. The study showed that silicone oil resulted in an underdosing to the tumor apex of 6.1% for I-125 and 7.5% for Pd-103 in the 15 mm plaque and 3.4% and 4.3% in the 23 mm plaque for I-125 and Pd-103, respectively. Taking a value of 85 Gy applied to the tumor apex in all scenarios, silicone oil reduced the dose to the inner sclera 90° from the plaque by 19–32% for the 15 and 23 mm plaques using I-125 and by 33–65% for the 15 and 23 mm plaques using Pd-103. It was found that Pd-103 plaque with silicon oil spared healthy tissues better than Pd-103 plaques alone or I-125 plaques with or without silicone oil. However, serious potential complications associated with the vitrectomy procedure should be borne in mind when considering such a method [49].

Lyons et al. described the use of the protective properties of silicone oil in patients. Five patients with medium- or large-sized melanomas were included in the study. All of them had at least one risk factor of radiation retinopathy. From 2011 to 2016, simultaneously with I-125 brachytherapy, they underwent a pars plana vitrectomy (PPV) with the administration of 1000 or 5000-centistoke (cSt) silicone oil and fine-needle aspiration biopsy (FNAB). One week after the first procedure, the plaque and silicone oil were removed. The dose for all patients was 85 Gy to the tumor apex. The follow-up time was between 12 and 56 months (median 45 months). The dose received by the macula ranged from 12.55 Gy to 141.5 Gy (median 43.17 Gy). Two patients whose tumors were located in the macular region, meaning that their maculas received the highest doses of radiation (141.5 Gy and 118.5 Gy), developed radiation retinopathy and their visual acuity deteriorated to counting fingers or hand motion during follow-up. Patients whose tumors were located in regions further from the posterior pole presented better visual acuity after treatment. Researchers connected this to a much lower dose of radiation received by the macula, which resulted not only from distance, but was also due to the protective properties of silicone oil. In the case of tumors located in the area of the macula, the possibilities of silicone oil cannot be utilized [50].

Shields et al. presented further results of a study based on prophylactic intravitreal injections of bevacizumab in patients with plaque-irradiated uveal melanoma. Prophylactic bevacizumab was administered between 2008 and 2018 to 1131 eyes with irradiated uveal melanoma at the time of plaque removal and as six further injections at 4-month intervals over 2 years. The study group was compared with the control group containing 117 eyes with irradiated uveal melanoma between 2007 and 2009 without prophylactic bevacizumab injections. This action was to prevent radiation retinopathy development and consequently to allow for maintaining better visual acuity. The mean follow-up was 40 months in the study group and 56 months in the control group. The group treated with bevacizumab demonstrated less OCT evidence of cystoid macular edema, less clinical evidence of radiation maculopathy, less clinical evidence of radiation papillopathy and better visual acuity at all points, including 12 months (median logMAR visual acuity (Snellen equivalent): 0.30 (20/40) vs 0.48 (20/60); mean difference, −0.28; 95% CI, −0.48 to −0.07; *p* = 0.02), 24 months (0.40 (20/50) vs 0.70 (20/100); mean difference, −0.52; 95% CI, −0.75 to −0.29; *p* < 0.001), 36 months (0.48 (20/60) vs 1.00 (20/200); mean difference, −0.49; 95% CI, −0.76 to −0.21; *p* = 0.003) and 48 months (0.54 (20/70) vs 2.00 (counting fingers); mean difference, −0.71; 95% CI, −1.03 to −0.38; *p* < 0.001). It must be noted that patients with OCT-evident macular edema at any time during follow-up were offered a therapeutic dose of anti-VEGF, including monthly intravitreal bevacizumab, ranibizumab, or aflibercept [41]. The achieved results of visual acuity in this and the previously mentioned studies encourage the continuation of research in this promising direction and perhaps the inclusion of this procedure in the standards of treatment [38,39,51].

The optimization of radiotherapy can be sought already at the planning stage, which is encouraged by the authors of the next study presented. As mentioned earlier, before using proton therapy, the patient first underwent a procedure of surgically suturing tantalum clips to the sclera. Measurements of the distance between the structures of the eyeball were also performed using ophthalmic imaging techniques, such as OCT, USG and fundus photography. Volumetric imaging—computer tomography (CT) or magnetic resonance imaging (MRI)—is usually not used in further computer planning. Via et al. proposed a different, alternative method of creating a model of the eyeball before treatment based on the image fusion of volumetric MRI and panoramic fundus photography. The researchers used the above method in 18 patients included in the study, who were treated in 2017–2020. The study showed that the combination of fundus photography and MRI to define tumor volumes reduced the mean discrepancies by almost 65% with respect to the MRI-only tumor definitions when compared with the conventionally planned target definition. Moreover, by using the proposed method, it was possible to include shallow sub-retinal tumor infiltration in the target volume definition, which was otherwise invisible on MRI. This method seems to provide greater personalization of therapy, and thus better accuracy, resulting in better local control and a lower number of post-radiation complications. However, the researchers emphasized that the use of the new, proposed method did not allow resignation from the use of tantalum tracers [52].

Alternative methods of treatment planning have also been sought for brachytherapy. Miras Del Rio et al. proposed a mathematical model that could be used for more accurate treatment planning. The method they proposed used a personalized model of the patient’s eye, taking into account tissue composition. The results were presented in terms of the dose delivered to real media, instead of the dose delivered to water, as was the case in previous planning. The calculations based on the proposed model of the dose received by the tumor and the structure of the eye differed, often significantly, from those obtained in the currently used standard methods. This led to a better understanding and possible implementation of new mathematical solutions for planning therapy in order to optimize it [53].

## 7. Discussions

Uveal melanoma is a huge threat to a patient’s life. Unfortunately, we are currently unable to effectively fight distant metastases to other organs, which, in a short time, lead to the death of the patient [11,12]. At the same time, owing to the efforts of doctors, medical physicists and other scientists, great progress has been achieved in the treatment of primary tumors using radiotherapy without the removal of the eyeball over the past several decades [1,3,4,18,19]. Unfortunately, radiotherapy also has its disadvantages, which include radiation complications often resulting in a diametric worsening of visual acuity, complete blindness of the treated eye or even the removal of the often-painful eyeball [9,11,31].

In this article, we focused on one of the post-radiation complications in the form of decreased visual acuity. This is a very important factor for the quality of life of patients after treatment, and its importance seems to be even greater when we remember that uveal melanomas also affect patients with one eye or with very low visual acuity in the other eye. However, we must not forget that the deterioration of vision is only partly due to radiation therapy. The impact of the tumor itself also contributes to it, e.g., through retinal detachment, vitreous hemorrhage and many other factors. We should also mention toxic tumor syndrome, which may occur when an irradiated, ischemic tumor releases cytokines. This can be prevented by tumor resection under the scleral flap (exoresection) or during PPV (endoresection) [54].

Comparing retrospective studies with each other has limited value because of the many differences usually occurring, ranging from the analyzed populations through different inclusion and exclusion criteria, different ranges of groups of BCVA acuity results, and ending with different clinical features and many other differences between studies. Even the comparison of visual acuity after therapy between groups treated with various types of radiotherapy within the same study is difficult due to different recommendations regarding the qualification of patients for different types of radiotherapy resulting from the location of the tumor, its size, diseases and the general condition of the patient [1,4,18]. Post-radiotherapy procedures, such as cataract surgery and anti-VEGF intravitreal injections that affect BCVA are also reported differently or not reported in different studies. Nevertheless, such comparisons also have significant value and, bearing in mind the limitations, we can draw valuable conclusions from them.

Bearing in mind the limitations mentioned above and comparing the research studies on visual acuity deterioration after Ru-106 brachytherapy, I-125 brachytherapy and proton beam therapy of uveal melanoma published at the turn of the 20th and 21st centuries [3,30,55,56] and in the last 4 years [9,11,21,31,33,34,35,37,38,39,41,42,44,49,50,51], we can conclude that the degree of visual impairment is comparable. Considering the extremely dynamic development of medicine and widely understood technology, one would like to achieve greater progress in this field. Various factors affecting the deterioration of vision after radiotherapy have been described [34,35,44]. Some of them, such as the distance of the tumor from the optic disc or macula, are unmodifiable. However, there are others, such as the dose of radiation absorbed by the macula, that we can modify. It seems to us that it is in these modifiable factors that we can look for better was to preserve vision.

A dose at the prescription point, which is the tumor apex, ranging from 70 to 100 Gy was recommended by the American Brachytherapy Society in 2014. Before the update, it was 85 Gy [57]. The research results of Fili et al. showed that there were no increased risks for choroidal melanoma-related mortality after brachytherapy with decreasing doses between 108.6 and 73.0 Gy [45]. These results raise the question of how much more we can limit the dose to the tumor apex in order to maintain its effectiveness in inactivating the tumor and limit its absorption by the surrounding tissues. While the administration of prophylactic intravitreal injections with an anti-VEGF after radiotherapy, proposed by Shields et al., does not seem to raise ethical doubts, the search for the minimum effective dose seems to carry a certain risk of exposing the patient to local recurrence and possible metastasis [51].

A method of protecting healthy tissues in the form of replacing the vitreous body with silicone oil proposed by Yang et al., Lyons et al. and other researchers suggests that further searching for substances that protect against radiation even better and, at the same time, are safer for the eye is needed [47,48,49,50]. However, serious potential complications associated with the vitrectomy procedure should be borne in mind when considering such a method.

Imaging fusion, proposed by Via et al. for proton therapy, seems to be extremely interesting and has great potential [52]. The greatest advantages seem to be the use of well-known and widely available imaging methods, and, additionally, owing to greater precision, not only the reduction of irradiation of healthy tissues, but also greater efficiency in tumor inactivation.

Additionally, a new mathematical model presented by Miras Del Rio et al. seems to be easy to implement if its superiority over the currently used one can be confirmed [53].

It is possible to introduce different modifications of a therapy to a single patient or to apply different improvements to different patients, depending on the clinical circumstances.

It is generally accepted that prospective, randomized trials have the highest scientific value. Meanwhile, the vast majority of studies on the treatment of uveal melanoma are retrospective studies. This is understandable due to the specificity of the disease and the related ethical dilemmas when planning potential prospective studies. It seems, therefore, that we should look for new and test the already proposed solutions of radiotherapy modifications, test greater personalization of therapy in order to reduce radiation complications while maintaining or even improving local tumor control and, if possible in the absence of ethical concerns, also conduct randomized prospective studies.

## 8. Conclusions

Radiotherapy, which is currently used in most patients with uveal melanoma, enables the preservation of the eyeball with the risks of metastasis and mortality comparable to those of enucleation. Unfortunately, it very often leads to a significant deterioration of visual acuity, which is caused, among other factors, by radiation complications. In recent years, new concepts to improve radiotherapy in order to increase its effectiveness and decrease the number of post-radiation complications have been developed. We should look for new and test the already existing radiotherapy modifications and try to introduce a greater personalization of therapy in order to reduce radiation complications while maintaining or even improving local tumor control.

## Figures and Tables

**Table 1:** Summary of the most important features of the analyzed therapies.

	Type of Radiation	Indications	Local Tumor Control (%)	Most Common Radiation Complications	Final VA of Less than 20/200 (%)
Brachytherapy Ru-106	Beta	Tumors up to 5 mm in height	59–98	Maculopathy, retinopathy, optic neuropathy, cataract	23–87 *
Brachytherapy I-125	Gamma	Tumors up to 10 mm in height	76–100	Maculopathy, retinopathy, cataract, neovascular glaucoma	23–87 *
Proton therapy	Beam of protons	Larger tumors with a height of more than 5 mm, narrow base of tumor, close to the optic nerve, ciliary body involvement of more than one clock hour, extrascleral extension	95	Maculopathy, retinopathy, cataract, optic retinopathy, rubeosis iridis	33–86 *

* The latest publications presented in this article differed from each other in many aspects. The differences concerned, e.g., patient groups, clinical parameters, visual acuity intervals, time of visual acuity testing and how it was presented. There were single studies, but also large systematic reviews. Considering these and other differences, we believe that comparing visual acuity between such different studies in one table may not be appropriate; therefore, we have included only approximate values in the table and strongly recommend the in-text summaries of each reviewed study.

## Data Availability

Not applicable.
